# Melanoma Differentiation-Associated Gene 5 (MDA5) Is Involved in the Innate Immune Response to *Paramyxoviridae* Infection In Vivo

**DOI:** 10.1371/journal.ppat.1000734

**Published:** 2010-01-22

**Authors:** Leonid Gitlin, Loralyn Benoit, Christina Song, Marina Cella, Susan Gilfillan, Michael J. Holtzman, Marco Colonna

**Affiliations:** 1 Department of Pathology and Immunology, Washington University School of Medicine, St. Louis, Missouri, United States of America; 2 Pulmonary and Critical Care Medicine, Department of Internal Medicine, Washington University School of Medicine, St. Louis, Missouri, United States of America; 3 Department of Cell Biology and Physiology, Washington University School of Medicine, St. Louis, Missouri, United States of America; University of North Carolina, United States of America

## Abstract

The early host response to pathogens is mediated by several distinct pattern recognition receptors. Cytoplasmic RNA helicases including RIG-I and MDA5 have been shown to respond to viral RNA by inducing interferon (IFN) production. Previous in vitro studies have demonstrated a direct role for MDA5 in the response to members of the *Picornaviridae*, *Flaviviridae* and *Caliciviridae* virus families ((+) ssRNA viruses) but not to *Paramyxoviridae* or *Orthomyxoviridae* ((−) ssRNA viruses). Contrary to these findings, we now show that MDA5 responds critically to infections caused by *Paramyxoviridae* in vivo. Using an established model of natural Sendai virus (SeV) infection, we demonstrate that MDA5^−/−^ mice exhibit increased morbidity and mortality as well as severe histopathological changes in the lower airways in response to SeV. Moreover, analysis of viral propagation in the lungs of MDA5^−/−^ mice reveals enhanced replication and a distinct distribution involving the interstitium. Though the levels of antiviral cytokines were comparable early during SeV infection, type I, II, and III IFN mRNA expression profiles were significantly decreased in MDA5^−/−^ mice by day 5 post infection. Taken together, these findings indicate that MDA5 is indispensable for sustained expression of IFN in response to paramyxovirus infection and provide the first evidence of MDA5-dependent containment of in vivo infections caused by (−) sense RNA viruses.

## Author Summary

The innate immune system possesses an array of sensory molecules which are purposed in detecting viral nucleic acids. Our understanding of how these molecular sensors detect viral nucleic acids continues to evolve. Herein, we demonstrate that MDA5, a member of the RIG-I-like receptor family, is involved in the detection of paramyxovirus infection in vivo. Specifically, MDA5 appears to trigger antiviral cytokines that inhibit paramyxovirus replication. In this regard, mice that are deficient in MDA5 are unable to express sustained levels of these cytokines and thus succumb to extensive viral propagation and disease. Our findings are largely discordant from previous in vitro studies using cultured cells, where it has been shown that RIG-I and not MDA5 is involved in the innate response to negative sense RNA viruses. Thus, our data provides strong evidence of MDA5-based detection of negative sense RNA viruses, and furthermore underscore the importance of organism-based analysis of the innate system.

## Introduction

Innate pathogen sensors detect viral products and respond by initiating a signaling cascade that leads to rapid anti-viral response involving secretion of type I IFNs (i.e. IFN-*α* and IFN-*β*) and inflammatory cytokines (i.e. IL-6 and TNF-*α*) [1]. In particular, type I IFNs restrict infection by inhibiting viral replication within cells and by stimulating the innate and adaptive immune responses. Once induced, secreted IFN-*α* and IFN-*β* bind to the IFN*α* receptor on the cell surface in an autocrine or paracrine manner. Activation of this receptor initiates the JAK/STAT signal transduction pathways [2],[3] and the expression of IFN-inducible genes [4]. These gene products increase the cellular resistance to viral infection and sensitize virally-infected cells to apoptosis [5]. In addition, type I IFNs directly activate DC and NK cells, and promote effector functions of T and B cells, thus providing a link between the innate response to infection and the adaptive immune response [6],[7].

Several viral sensors have been identified that belong to the Toll-like receptor (TLR) and RIG-I like receptor (RLR) families [8]. TLRs are expressed on the cell surface and/or in endosomal compartments [9]. TLR3 recognizes double stranded RNA (dsRNA), a molecular pattern associated with replication of single stranded RNA (ssRNA) viruses as well as the genomic RNA of dsRNA viruses [10]. TLR7 and TLR8 recognize ssRNA [9],[11],[12], whereas TLR9 recognizes unmethylated CpG-containing DNA [13]. RLRs are cytoplasmic proteins that recognize viral nucleic acids that have gained access to the cytosol [14]–[19]. The RLR family consists of three known members: retinoic acid-inducible gene I (RIG-I), melanoma differentiation-associated gene 5 (MDA5), and LGP2. RIG-I and MDA5 both contain a DExD/H box helicase domain that binds dsRNA, a C-terminal domain and two N-terminal caspase recruitment domains (CARDs) that are involved in signaling [8],[17],[20],[21]. LGP2 contains a helicase domain but lacks the CARDs, and its precise contribution to antiviral signaling remains ambiguous [17],[22].

Though RIG-I and MDA5 share common downstream signaling via activation of IPS-1 (also called MAVS, VISA or Cardif) and IRF3 [23]–[26], these helicases exhibit distinct substrate specificity. In this regard, RIG-I has been shown to preferentially recognize ssRNA that is phosphorylated at the 5′ end [27],[28] and dsRNA molecules which are relatively short [29]–[31]. In contrast, MDA5 recognizes long dsRNAs but does not discern 5′ phosphorylation[30],[32],[33]. This distinct ligand preference has been shown to confer specific recognition of individual viruses: RIG-I has been shown to detect Influenza A and B viruses, paramyxovirus, vesicular stomatitis virus (all (−) ssRNA virues) and some Flaviviruses ((+) ssRNA viruses including Japanese encephalitis virus, Hepatitis C virus and West Nile virus)[16],[33],[34]. In comparison, MDA5 has been shown to selectively detect (+) ssRNA viruses including picornaviruses (encephalomyocarditis virus, Mengo virus and Theilers virus) [32],[33], *Caliciviridae* (murine norovirus-1) [35], and *Flaviridae* (West Nile Virus and Dengue Virus) [34],[36]. Accordingly, it is believed that the presence of different classes of sensors may reflect the need for multiple mechanisms to effectively control the wide variety of viral pathogens.

Paramyxoviruses are (−) ssRNA viruses that are responsible for a number of human diseases including those caused by measles, mumps, parainfluenza virus and respiratory syncytial virus (RSV). Importantly, infections caused by paramyxoviruses are the most frequent cause of serious respiratory illness in childhood and are associated with an increased risk of asthma [37],[38]. Sendai virus (SeV) is a murine parainfluenza virus which causes an acute respiratory disease in mice that resembles severe paramyxoviral bronchiolitis found in humans following RSV infection [39]. To date, RIG-I is the only dsRNA sensor that has been implicated in the veritable detection of paramyxoviruses [33],[40]. The importance of RIG-I in the containment of SeV infection is underscored by capacity of SeV C proteins to directly antagonize RIG-I signaling [41] in addition to their ability to inhibit IFN signal transduction [42],[43]. However, paramyxovirus-encoded V proteins are known to directly interfere with MDA5 function by blocking binding of dsRNA [14],[44], thus implicating MDA5 in the containment of paramyxovirus infection as well. In addition, SeV defective interfering (DI) particles have been shown to engage MDA5 in vitro [45], though the in vivo relevancy of this detection mode is unknown. Thus, to determine whether MDA5 functions during natural infection with paramyxovirus in vivo, we assessed mice deficient in MDA5 (MDA5^−/−^ mice) following respiratory tract infection with SeV.

## Results

### Infection with SeV causes increased morbidity and mortality in MDA5^−/−^ mice

**Figure 1:**
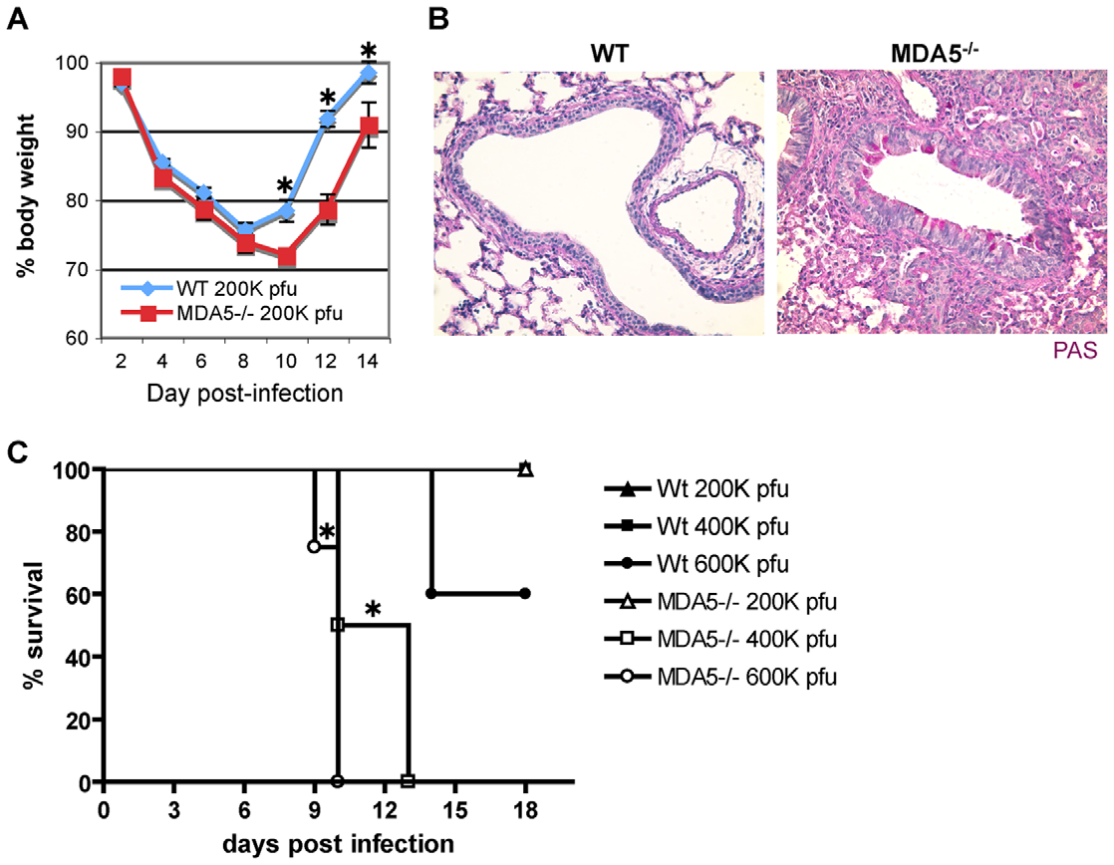
Infection with SeV causes increased morbidity and mortality in MDA5^−/−^ mice. WT and MDA5^−/−^ mice were infected with 200K pfu SeV and assessed for A) loss of body weight over the PI period and B) mucus production (PAS reactivity). C) WT and MDA5^−/−^ mice were infected with 200K, 400K and 600K pfu SeV and assessed for viability. N = 4–16 mice, error bars refer to SEM, * P≤0.05.

In order to assess an in vivo role for MDA5 in containment of paramyxovirus infection, we infected MDA5^−/−^ mice with Sendai virus (SeV). Mice on a C57BL/6 (B6) background were selected for these experiments as the 129 strain is lethally susceptible to SeV at extremely low inocula [46], thus prohibiting assessment of loss of MDA5 function on this background. A dose of 200,000 pfu was administered to mice by intranasal delivery, an infection method that typically results in acute, non-lethal bronchiolitis in B6 mice. As a gross determinant of virus-induced morbidity, % body weight for infected WT and MDA5^−/−^ mice was monitored for 2 weeks post infection (PI). Though essentially identical % weight loss values were observed up until day 8 PI; onwards, weight loss in MDA5^−/−^ mice was significantly more severe (p<0.05) (Figure 1A). Correspondingly, histological analysis of lung sections obtained from day 12 PI MDA5^−/−^ mice revealed consolidation of the lung parenchyma as well as notable PAS-positive airway cells, an indication of mucus hyper-secretion (Figure 1B). Severe histopathology was not observed in the lung sections obtained from control mice at this time point. In addition, we compared survival following increasing inocula of SeV (Figure 1C). Though MDA5^−/−^ mice were not susceptible to the 200K pfu SeV dose, MDA5^−/−^ mice fully succumbed to 400K and 600K pfu SeV, between 9–14 days PI. In contrast, control mice were fully resistant to the 400K pfu dose, though 40% mortality was observed for controls infected with the 600K dose. Thus MDA5^−/−^ mice exhibit enhanced morbidity and susceptibility to SeV infection relative to control mice.

**Figure 2:**
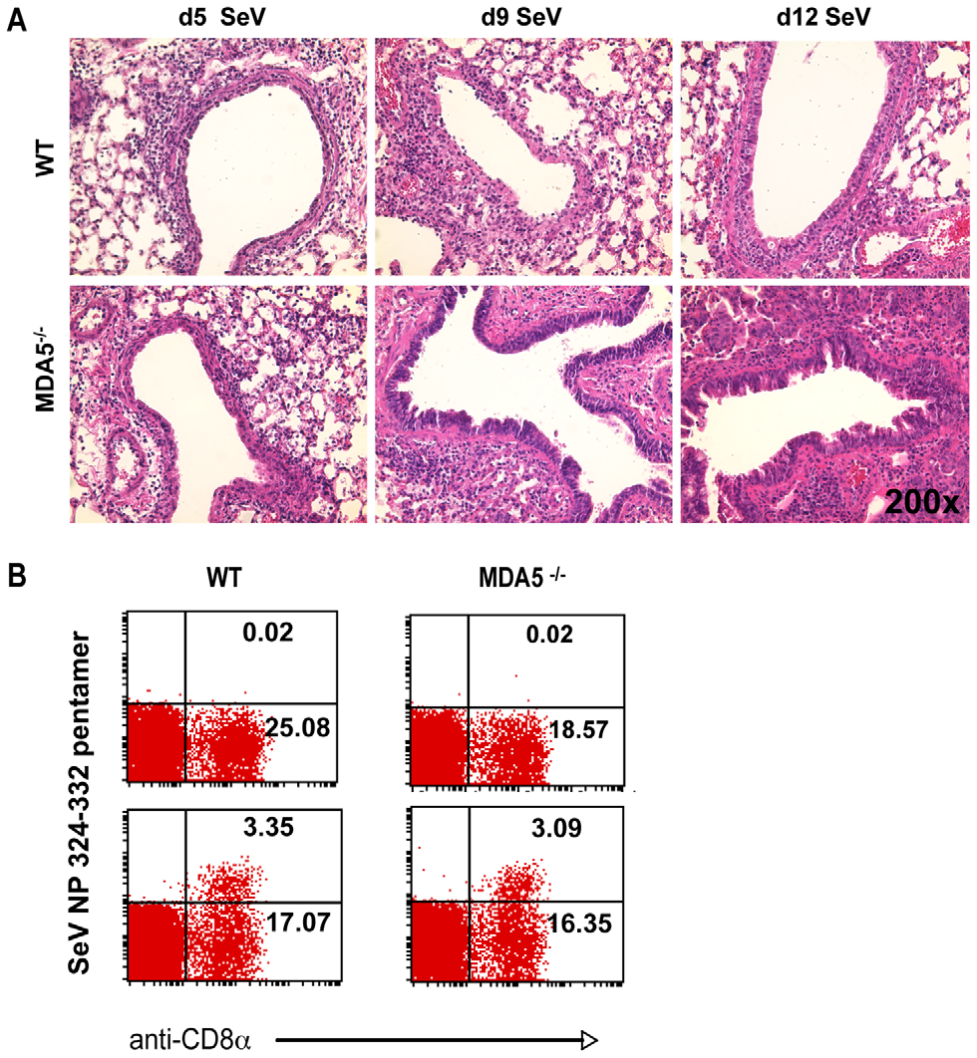
Increased histopathology in MDA5^−/−^ mice. A) H&E micrographs of lung sections obtained from WT and MDA5^−/−^ mice infected with 400K pfu SeV on d5, d9, d12 PI. B) FACS analysis of lymphocytes derived from the lungs of WT and MDA5^−/−^ mice, *uninfected (top panels) and d5 post infected (bottom panels)* stained with anti-CD8 and H-2K^b^: FAPGNYPAL pentamer.

To more fully assess SeV susceptibility, we extended our analysis of the histological changes seen in the lungs of SeV-infected MDA5^−/−^ mice. H&E stained sections obtained from day 2 PI (not shown) and day 5 PI (Figure 2A) lungs demonstrated similar patterns of bronchiolitis, though peribronchiolar lymphoid cuffing that formed in the lungs of control mice appeared moderately thicker and more densely populated than those of MDA5^−/−^ mice (Figure 2A). FACS analysis of lung-derived leukocytes at d2, d5, and d8 PI revealed no significant differences in lymphoid and myeloid subpopulations (neutrophils, cDC, macrophage and alveolar macrophage; data not shown). Significantly, at d5 and d8 PI, FACS analysis revealed equal relative numbers of lymphoid subpopulations (CD3^+^, CD19^+^ and NK1.1^+^); CD69 expression profiles on these subsets were comparable between strains (data not shown). By d8-9 PI, significant pathology was observed in the lungs of SeV-infected MDA5^−/−^ mice (Figure 2A), despite the fact that comparable numbers of SeV-specific CTL were generated in both strains at this time point (Figure 2B). Grossly, lungs dissected from SeV-infected MDA5^−/−^ mice exhibited enhanced areas of hemorrhage relative to control lungs (data not shown). Microscopic analysis revealed epithelial cells that were notably hyperplastic with abundant micropapillary projections. Additionally, severe bronchointerstitial pneumonia was observed, with alveolar walls adjacent to affected airways thickened and congested with chronic inflammatory cell infiltrates and hyperplastic type II pneumocytes, a lung injury pattern consistent with SeV susceptibility [46],[47]. In comparison, sections obtained from control mice at these later time points exhibited moderate changes to the airway epithelium and mild interstitial infiltration (Figure 2A).

### MDA5^−/−^ mice demonstrate increased susceptibility to SeV propagation

**Figure 3:**
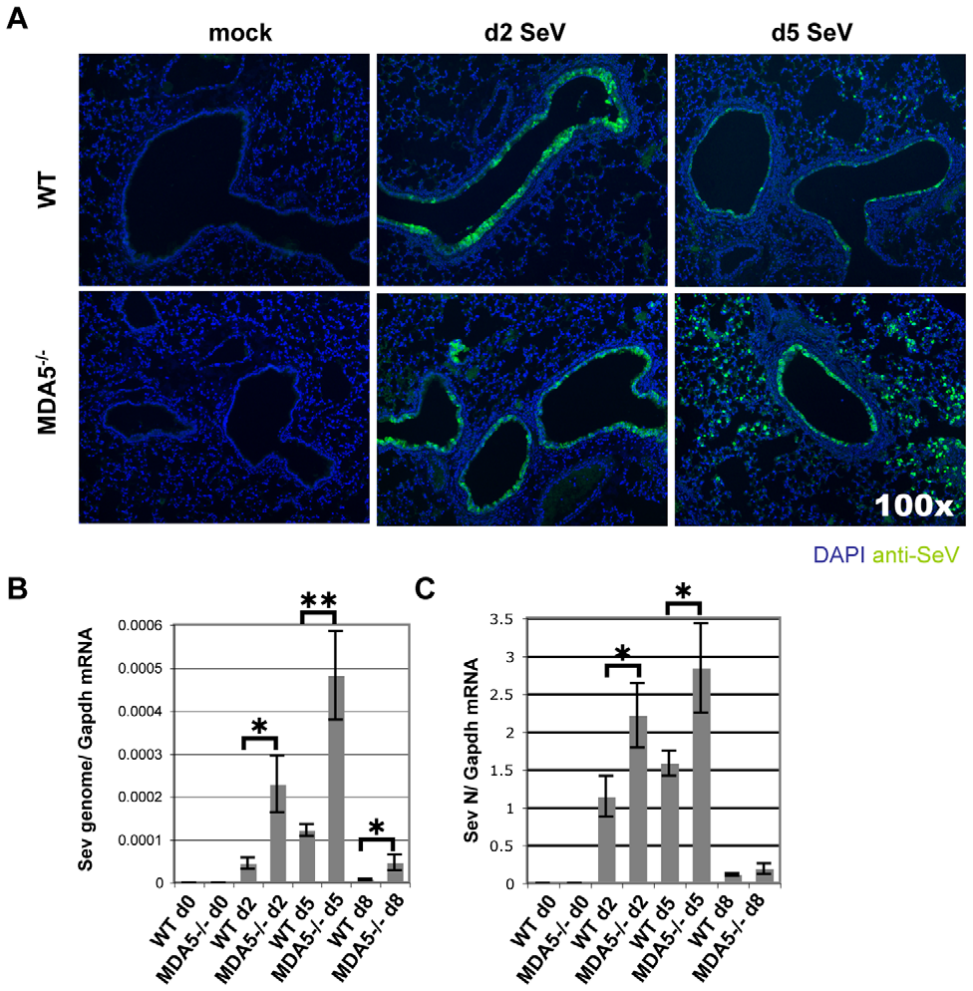
SeV replication is enhanced in MDA5^−/−^ mice. WT and MDA5^−/−^ mice infected with 200K pfu SeV were assessed for A) SeV replication by IF detection of SeV antigens and by real time PCR analysis of B) SeV genome and C) SeV N gene expression. N = 4, error bars refer to SEM, * P<0.05; ** P<0.005.

As susceptibility to SeV infection correlates with increased viral burden [48], we next assessed viral replication in wild type and MDA5^−/−^ mice using a combined approach of real-time PCR analysis and specific staining for SeV antigens. Initially, at d2 PI, IF staining of viral antigens in lung sections appeared comparable between the two strains. By d5 PI, SeV antigens exhibited restrained expression in the airways of control mice (Figure 3A top of panel). In contrast, the bronchioles of MDA5^−/−^ mice remained notably positive for SeV antigens at this time point (Figure 3A bottom of panel). More striking however, was the observation that parenchyma tissues proximal to infected airways stained conspicuously for SeV antigens in MDA5^−/−^ mice at d5 PI. In SeV resistant strains of mice, SeV infection is typically restricted to the mucociliary epithelium of the conducting airways, including the trachea, bronchi and bronchioles [49],[50]. Viral replication that extends to the alveolar spaces is a feature commonly seen in susceptible strains of mice [51]. Accordingly, this pattern of infection supports a role for MDA5 in controlling the replication of SeV during in vivo infection.

To confirm this finding, we measured viral RNA levels from WT and MDA5^−/−^ mice infected with 200K pfu SeV using real-time PCR analysis. Assessment was made using primer/probe sets designed to amplify SeV genome (3′ untranslated region) and SeV N gene (genomic and transcript) (Figure 3B and C). Using this strategy, an approximate 5 fold increase in SeV genome copy number/*Gapdh* mRNA was detected on d5 PI, though significant differences were also observed on d2 and d8 PI. Analysis of N gene revealed ∼2 fold increase in expression on days 2 and 5, though there were no significant differences by d8 PI. Thus it appears that MDA5 contributes in part to the containment of SeV replication in vivo.

### Cytokine response to SeV infection is altered in MDA5^−/−^ mice

**Figure 4:**
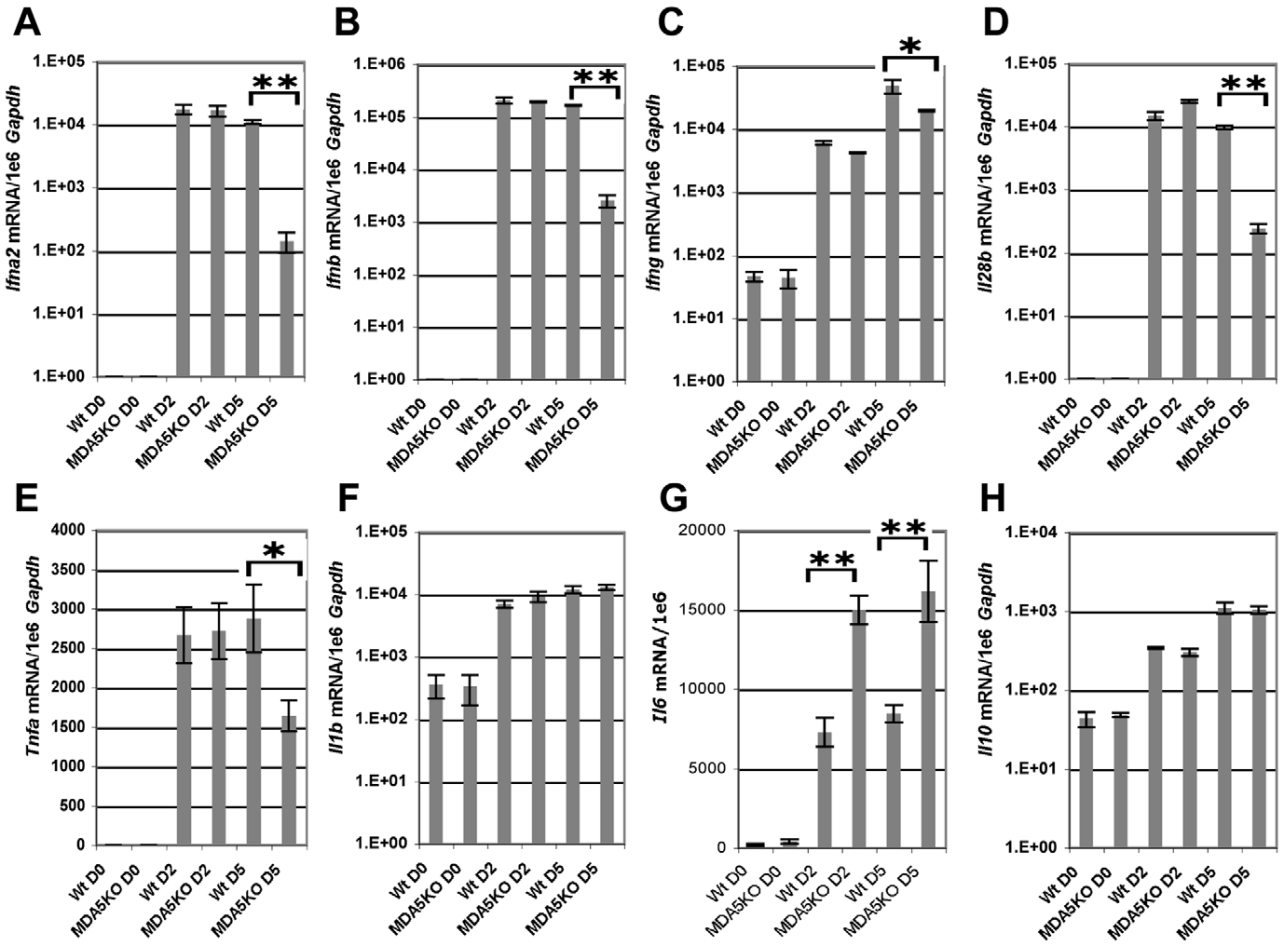
MDA5 is required for sustained expression of cytokines in response to SeV infection. Real time PCR analysis of whole lung homogenates obtained from WT and MDA5^−/−^ mice infected with 200K pfu SeV for expression levels of A) *Ifn-α2*, B) *Ifn-β*, C) *Ifn-γ*, D) *Il-28b*, E) *Tnf-α*, F) *Il-1β*, G) *Il-6 and* H) *Il-10* mRNA. N = 4, error bars refer to SEM, * P<0.05, ** P<0.00001.

Though SeV is a potent inducer of type I IFN in the mouse, functioning via several distinct pathways, it possesses several mechanisms by which it can counteract the IFN response. Despite this property, induction of IFN expression [14],[52], particularly type I and II, is critical in the containment of SeV infection in vivo as underscored by the profound SeV susceptibility seen for mice deficient in STAT1^−/−^ mice [53]. As MDA5 is known to induce expression of type I IFN in vitro in response to polyI:C stimulation and viral infection [17], we sought to directly assess the ability of MDA5^−/−^ mice to express IFN in response to SeV infection. In this regard, WT and MDA5^−/−^ mice were infected with 200K pfu SeV and subsequently assessed for cytokine expression by real-time PCR analysis over the acute period. While both strains demonstrated comparable mRNA levels at d2 PI, type I IFN expression was dramatically dampened in MDA5^−/−^ mice at d5 PI (Figure 4A and B). Unexpectedly, significant decreases in expression of *Ifn-γ*, *Il-28b (Ifn-λ3*) and *Tnf-α* mRNA were also observed in the lungs of MDA5^−/−^ mice compared to the WT cohort, with the most dramatic difference observed for *Il-28b* mRNA expression (Figure 4C, D and E). In contrast, *Il-1β*, and *Il-10* mRNA levels were not significantly different across strains, though the levels of *Il-6* mRNA was markedly increased in MDA5^−/−^ mice following infection (Figure 4F, G and H). Accordingly, MDA5 appears to control the expression of SeV-induced anti-viral cytokines, particularly type I, II and III IFNs, during the late acute period (d5 PI), but does not appear to be involved during the immediate early response. Importantly, the decrease in IFN expression coincides with expanded viral propagation in the MDA5^−/−^ mice, suggesting that reduced IFN expression during this time point accounts for the corresponding increased viral burden.

**Figure 5:**
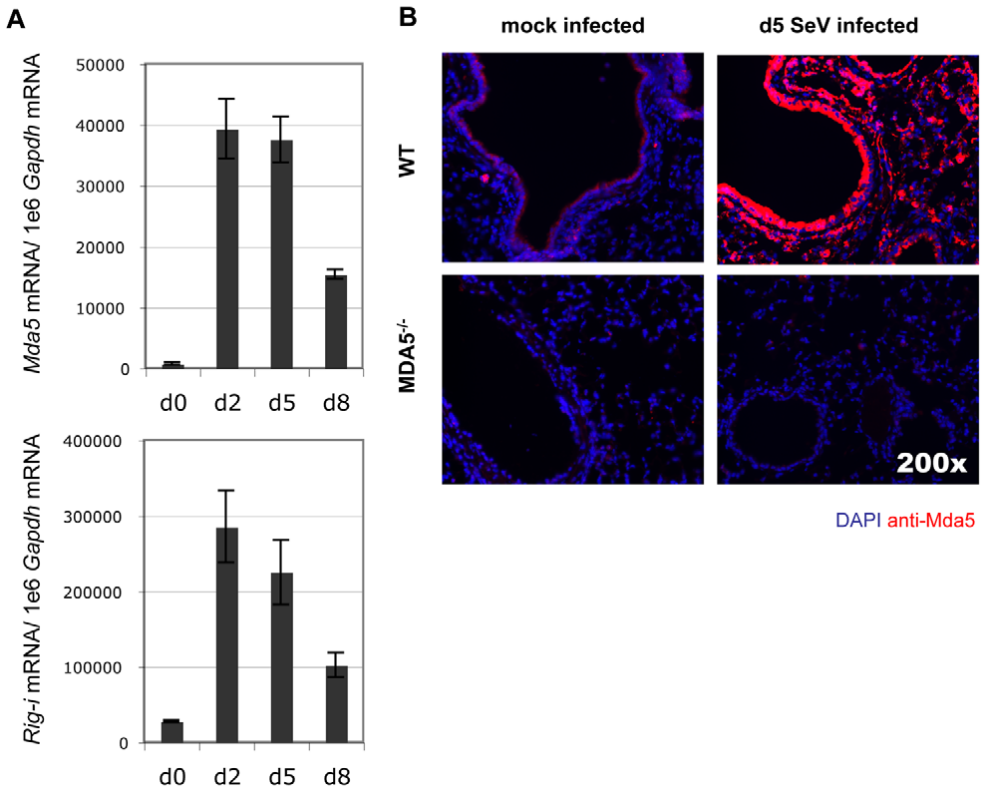
Infection with SeV results in induction of antiviral sensor expression. A) Analysis of *Mda5* and *Rig-I* mRNA expression in WT mice during the acute SeV infection period as determined by real-time PCR analysis. B) Micrographs taken of lung sections obtained from WT and MDA5^−/−^ mice infected with 200K pfu SeV and stained for MDA5 expression. N = 4, error bars refer to SEM, * P<0.05.

Induction of IFN expression transactivates expression of a number of IFN response genes through a signal transduction cascade involving JAK/STAT activation. MDA5 and RIG-I are among the genes induced by IFN signaling in vitro [20]. To determine the expression profile of MDA5 and RIG-I in the airways of mice infected with SeV, mRNA was measured by real-time PCR analysis from whole lung homogenates obtained from WT mice infected with 200K pfu SeV. Expression of *Mda5* and *Rig-i* mRNA was significantly increased at d2 and d5 PI, though the levels began to decline by d8 PI (Figure 5A). Lastly, to determine the tissue distribution of MDA5 expression, lung sections from d5 PI mice were stained with anti-MDA5 polyclonal antibodies. Visualization of MDA5-specific staining was performed using tyramide-based amplification. IF microscopic analysis of affected airways revealed a pattern of MDA5 expression that was primarily restricted to the airway epithelium, though expression was also detected in cells of the proximal interstitum, in particular, in cells that appeared to resemble type II pneumocytes and alveolar macrophage (Figure 5B). Sections from MDA5^−/−^ mice did not stain for MDA5, confirming the specificity of anti-MDA5 staining. Accordingly these findings indicate that MDA5 is induced following SeV infection and that the lack of expression in MDA5^−/−^ mice accounts for the phenotype described at the later time point.

## Discussion

Our understanding of innate immune factors that recognize and respond to pathogens has greatly expanded over the last decade. A major component of the RNA virus detection system in mammals involves members of the RLR family, including RIG-I, MDA5, and LGP2 [1]. Elucidating a role for the RLRs in virus-induced IFN production has been facilitated by the availability of RIG-I^−/−^ and MDA5^−/−^ mice [32],[33]. Initial observations using embryonic fibroblasts and bone marrow derived DCs generated from these mice revealed striking phenotypes including a failure to produce IFN in response to a wide cross-section of viruses and nucleic acids, and an inability to contain viral replication. Specifically, MDA5 was found to be the sole receptor for picornaviruses and caliciviruses ((+) ssRNA viruses) [32],[33],[35], whereas RIG-I was described as the receptor for (−) ssRNA viruses such as paramyxoviruses and orthomyxoviruses, as well as for (+) ssRNA viruses belonging to the *Flaviridae* family [16],[33]. However, our understanding of these virus recognition systems in vivo is limited, in part because RIG-I^−/−^ mice die perinatally.

The precise molecular patterns of virus replication recognized by RIG-I and MDA5 are still not fully clear. Initially, a mimic of viral dsRNA, polyI:C, was found to bind and activate RIG-I. However, ensuing research identified 5′-triphosphate-linked ssRNA as the major RIG-I inducer [27],[28]. Furthermore, in vitro data obtained using knockout mice suggested in fact that MDA5, and not RIG-I, recognizes polyI:C, thereby formulating a recognition model whereby RIG-I recognizes short 5′-triphosphorylated RNAs, while MDA5 recognizes dsRNA structures irrespective of the 5′ cap [8], [30]–[32]. However, more thorough dissection of the helicase binding function and activation process has recently determined that the picture is indeed more complex than previously thought [29],[40]. In this regard, both helicases have been shown to recognize dsRNA, in a manner that is likely dependent on its length, while RIG-I demonstrates the added ability to respond to 5′-triphosphate ssRNA products. To complicate these paradigms, there is increasing evidence that viruses have evolved various properties aimed at antagonizing or degrading viral sensors. Thus, our understanding of viral recognition by the RLR helicases is evolving.

With respect to molecular sensing of paramyxovirus infection, both 5′-triphosphorylated ssRNA and long dsRNA species are likely present in SeV-infected cells, thereby implicating both MDA5 and RIG-I in the antiviral sensing process. However, in vitro studies concur that cultured embryonic fibroblasts and bone marrow-derived DC cells detect SeV RNA chiefly through RIG-I, whereas MDA5 and TLR3 are dispensable [33],[34],[41],[54]. TLR7 and TLR8 in myeloid cells have also been shown to recognize SeV RNA in vitro as well [55]. Regardless, it cannot be excluded that in vivo, other RNA sensors, including MDA5, may contribute, at least in part, to anti-SeV responses. Indeed, a recent study by Yount et al. has demonstrated that MDA5 can detect SeV DI particles in vitro [45]. The relevancy of this recognition system in vivo is uncertain; certainly in our hands, using SeV/52, which has a limited ability to form DI particles, as per PCR-based analysis (data not shown), MDA5 appears to exert a significant effect on viral containment. Most importantly however, SeV encodes a V protein that specifically binds to and blocks MDA5 signaling in vitro [14],[44]. Thus, it is possible that MDA5 does, indeed, detect SeV in vitro, but that it is functionally curtailed by the V protein in this circumstance. Interestingly, in our hands, in vivo infections using SeV with V protein deletion resulted in no real effect on mortality or type I IFN induction across strains (data not shown), likely explained by the fact that deletion of V protein in SeV markedly attenuates virulence and pathogenicty in vivo [56].

While initial characterization of MDA5-deficient cells has not supported a role for MDA5 in containment of SeV, these studies have been limited to observations made in cultured embryonic fibroblasts and in vitro-derived dendritic cells; populations which are not primary targets for SeV replication in the course of the natural infection. Rather, SeV replication mostly occurs in the airway epithelium of the conducting airways [49],[50]. For these reasons, we hypothesized that SeV propagation would be sensitive to the MDA5 status of the host in the context of an in vivo infection. Indeed, the epithelial cells constitutively express MDA5 at low levels and subsequently up-regulate expression in response to SeV (Figure 5B), a finding that supports the relevance of this RNA helicase to SeV and other airborne infections. Interestingly, MDA5 deficiency did not influence the composition of the inflammatory infiltrate (data not shown), implying that the immune defect is largely restricted to the airway epithelium, the site of viral replication. This is compatible with our earlier findings using STAT-1^−/−^ chimeras, wherein we observed that loss of IFN response in the stromal compartment alone accounted for the immune deficiency to SeV [53]. We therefore sought to further assess the significance of MDA5 in the control of SeV infection in vivo. In this regard we have demonstrated that MDA5 controls SeV replication and spread through induction of type I IFNs, but that this effect appears late (d5 PI), as IFN gene transcription is not impaired on d2 PI (Figure 4). It is likely that the initial IFN response is sufficient to initiate a range of immune responses, such that the late reduction in IFN transcripts results only in a 2–3 fold change in LD_50_ (Figure 1). Whether this specific IFN pattern remains true for other viruses as well remains to be tested. This surprising collapse of the host type I IFN response at d5 PI is accompanied by parallel decreases in the level of *Il-28b* and *Tnf-α* expression (Figure 4), and, curiously, decreased *Ifn-γ* transcript levels. This later observation may reflect a selective role for MDA5 in the induction of IFN-γ expression by NK cells. Lastly, the MDA5 status does not appear to influence the levels of IL-1β, or IL-10 or the ability of the host to mount a virus-specific CTL response. However, the levels of *Il-6* mRNA in whole lung homogenates derived from d2 and d5 PI MDA5^−/−^ was markedly increased, suggesting the induction of compensatory mechanisms in the context of MDA5 deficiency that could potentially account for the enhanced morbidity and mortality seen in the MDA5^−/−^ mice.

An additional concern raised by these data is the relative contribution of MDA5 and RIG-I in the response to virus. In light of the existing literature [33],[40], it seems likely that RIG-I is responsible for the normal IFN response to SeV early in the infection. Indeed, as depicted in Figure 5A, RIG-I is strongly induced early on during infection. Why the later IFN response depends on MDA5 is not known. MDA5 is encoded by an IFN-upregulated transcript, and it remains possible that it is the accumulation of MDA5 that allows for the subsequent MDA5-dependent IFN response on d5 PI. Yet other IFN-induced genes, notably RIG-I, are also upregulated by IFN, which should provide additional antiviral protection in vivo. Interestingly, SeV encodes a nested set of C proteins that have been shown to impede IFN signaling through direct inhibition of STAT signaling [41],[42] and which are also known to strongly antagonize RIG-I function [41]. Furthermore, SeV-V proteins have been shown to have direct inhibitory effects on both MDA5 and RIG-I signaling [41],[44]. Thus it remains possible that the effects of SeV V and C proteins have an accumulative effect on RIG-I function that essentially overwhelms this sensor at d5 PI, and that in this context, MDA5 plays an essential role in containment of SeV. Since assessment of the relative contribution of RIG-I and MDA5 in containment of SeV infection in vivo is not possible, a possible next step in assessing the importance of MDA5 function would involve assessment in MDA5^−/−^ and IPS-1^−/−^ strains.

We envision several possibilities that could potentially explain this dramatic effect of MDA5. The first is that, in the absence of MDA5, the balance between virus replication and the IFN response is disrupted sufficiently, such that by d5 PI, virus replication has overwhelmed the response in a qualitative fashion –presumably through direct cytotoxic effects or via the overproduction of immunosuppressive C proteins. This possibility is supported by the fact that SeV is replicating to higher levels in the MDA5^−/−^ lung already by d2 PI (Figure 3B). Indeed, in support of this hypothesis, we observe a striking increase in SeV replication that spreads extensively into the interstitium of MDA5^−/−^ lungs compared to controls. Another possible explanation, which we have not assessed, is an apoptotic response potentially mediated by MDA5. In this scenario, MDA5 would instruct or sensitize infected cells to commit suicide so as to shut down viral replication in infected cells. Indeed, ectopic expression of MDA5 in a melanoma cell line has been shown to inhibit colony formation, presumably through induction of apoptosis [20], and IPS-1 overexpression induces cell death, as well [57]. In fact, SeV-dependent apoptotic signaling requires IRF3 [58]. In the case of MDA5 deficiency, loss of pro-apoptotic activity could lead to a robust increase in viral replication and enhanced IFN blockade through overexpression of SeV C proteins. This possibility is favored by the fact that, despite a normal IFN response on d2 PI (Figure 4), the virus is found to be replicating at higher titers (Figure 3B).

It seems likely that the inability of the MDA5^−/−^ animals to sustain an IFN response leads to increased viral replication and dissemination on d5 PI, thus causing significantly higher morbidity and mortality in the knockout cohort (Figure 1). It is important to note, however, that the effects of MDA5 deficiency on SeV replication are much milder than expected if MDA5 were the sole target of the V protein. Indeed, SeV V mutants (SeV-ΔV) are severely attenuated; replication is demonstrably abrogated in the lungs by d2 PI [56]. IRF3 deficiency of the host restores SeV-ΔV pathogenicity, suggesting that the mutant virus acts by blocking IRF3 signaling [59]. Yet disease caused by SeV in MDA5^−/−^ mice is milder than the disease seen in the IRF3^−/−^ animals. Consequently, we believe that the V protein must have additional targets besides MDA5. In this regard, it has recently been demonstrated that *Lgp2* encodes a helicase epitope that is akin to the MDA5 helicase, the portion of MDA5 that binds paramyxovirus V proteins [60], thereby suggesting that LGP2 may be an additional V protein target. In this case, a MDA5-LGP2 double knockout mouse may potentially phenocopy the IRF3 mutation in its response to SeV infection.

Taken together, our findings demonstrate that MDA5 significantly contributes to the response to paramyxovirus and constitute the first in vivo demonstration of MDA5 activity against a negative-strand virus. As such, it appears likely that MDA5 has a wider specificity as a viral nucleic acid receptor than initially believed, and that the initial clear-cut cases of either MDA5 or RIG-I being the sole receptor for a given virus will prove to be exceptions rather than rules when studied in the context of in vivo infections.

## Materials and Methods

### Mouse generation, maintenance and infection

Control C57BL/6J (B6) mice used in these experiments were purchased from JAX. MDA5^−/−^ mice [32] were backcrossed onto the B6 background to 99.9% congenicity. For in vivo SeV infection, Sendai/52 Fushimi strain was instilled intranasally into deeply anesthetized mice and at the indicated time points, mice were humanly sacrificed for harvest of lung tissue. Virus was purchased from the ATCC and subject to two rounds of in vitro plaque purification in Vero cells to eliminate the presence of DI particles. A clone thus identified was then subject to a single round of amplification in embryonated chicken eggs following inoculation of ∼1000 PFU. 24–36 hr post inoculation, SeV was isolated from the allantoic fluids and diluted in phosphate-buffered solution to generate a viral stock that was subsequently characterized on the basis of in vivo infectious properties. Calculation of PFU was performed by standard plaque assay using either Vero E6 cells or LLC-MK2 cells. Importantly, propagation under these conditions does not favor the formation of DI particles, a process that occurs most frequently when virus is repeatedly passaged at high MOI. Indeed, PCR analysis of stock virus indicated the absence of DI genomes. The methods for mice use and care were approved by the Washington University Animal Studies Committee and are in accordance with NIH guidelines.

### FACS

Single cell lung suspensions were made from minced lung tissue subjected to collagenase/hyaluronidase/DNAse I digestion. Staining of surface markers was performed using FcR block and fluorochrome-conjugated mAbs. To immunophenotype the immune infiltrate, specific combinations of mAbs were chosen which discern granulocytes (Ly6G^+^), macrophages (F4/80^+^), cDC (CD11c^+^F4/80^−^Siglec-H^−^), pDC (Siglec-H^+^ CD11c^mid^), NK cells (NK1.1^+^NKp46^+^), T cells (CD3^+^CD4^+/−^CD8^+/−^) and B cells (CD19^+^). SeV-specific PE-labeled pentamer K^b^:FAPGNYPAL (NP 324-332) was purchased from Proimmune; cells were stained with CD8 and counterstained with propidium iodide, F4/80 and CD19 to eliminate background. Activation status was determined using specific mAbs for MHC-II, NKG2D and CD69. Samples were acquired on a FACScalibur (BD Biosciences) and analyzed using Cellquest software.

### Analysis of mRNA and virus-specific RNA

RNA was purified from lung homogenate using Trizol Reagent (Invitrogen). RNA was treated with RNAse-free DNAse I (Ambion) to eliminate genomic DNA. RNA was converted to cDNA using the High-Capacity cDNA Archive kit (Applied Biosystems). Target mRNA and viral RNAs were quantified by real-time PCR using specific fluorogenic probes and primers and the Fast Universal PCR Master Mix system (Applied Biosystems). Primer sets and probes for mouse *Ifn-α2* (Mm00833961_s1), *Ifn-β* (Mm00439552_s1), *Ifn-γ* (Mm00801778-m1), *Il-28b* (Mm00663660_g1), *Tnf-α* (Mm00443259_g1), *Mda5* (Mm00459183_m1), *Il-1β* (Mm00434227_g1), *Il-6* (Mm00446190_m1), *Il-10* (Mm00439616_m1) mRNA and SeV genome and *Gapdh* mRNA were purchased from Applied Biosystems. Samples were assayed on the 7500 Fast Real-Time PCR System and analyzed using the 7500 Fast System Software (Applied Biosystems). Levels of specific gene expression were standardized to *Gapdh* mRNA expression levels.

### Histology

Lungs were perfused and fixed with 4% paraformaldehyde. Tissue was embedded in paraffin, cut into 5 um sections and adhered to charged slides. Sections were deparaffinized in Citrosolv (Fisherbrand), hydrated, and in the case of IF-microscopy, treated to heat-activated antigen unmasking solution (Vector Laboratories, Inc). H&E and PAS sections were visualized by brightfield microscopy. Expression analysis was performed by IF using chicken polyclonal anti-SeV (Jackson ImmunoResearch Laboratories, Inc) and rabbit polyclonal anti-mouse MDA5 (Axxora Life Sciences, Inc). Biotinylated secondary antibodies were purchased from Vector Laboratories, Inc). SeV and MDA5 expression was visualized using tyramide-based signal amplification with Alexa Fluor 488 or 594 fluorochromes (Invitrogen). Slides were counterstained with DAPI mounting media (Vector Laboratories, Inc). Microscopy was performed using an Olympus BX51 microscope.

### Statistical analyses

Real-time PCR data was analyzed using an unpaired Student's t-test. If variances were unequal, Welch's correction was applied. Charted values represent mean ± SEM. Survival statistics were determined using by Kaplan-Meier analysis of paired cohorts. P values below 0.05 were regarded as being significant for all analyses. Experiments were repeated a minimum of three times.

## References

[1] Kawai T, Akira S (2008). Toll-like Receptor and RIG-1-like Receptor Signaling.. Annals of the New York Academy of Sciences.

[2] Kotenko SV, Pestka S (2000). Jak-Stat signal transduction pathway through the eyes of cytokine class II receptor complexes.. Oncogene.

[3] Schindler C, Darnell JE (1995). Transcriptional Responses to Polypeptide Ligands: The JAK-STAT Pathway.. Annual Review of Biochemistry.

[4] de Veer MJ, Holko M, Frevel M, Walker E, Der S (2001). Functional classification of interferon-stimulated genes identified using microarrays.. J Leukoc Biol.

[5] Stetson DB, Medzhitov R (2006). Type I Interferons in Host Defense.. Immunity.

[6] Braun D, Caramalho I, Demengeot J (2002). IFN-{alpha}/{beta} enhances BCR-dependent B cell responses.. Int Immunol.

[7] Tough DF (2004). Type I interferon as a link between innate and adaptive immunity through dendritic cell stimulation.. Leukemia & Lymphoma.

[8] Saito T, Hirai R, Loo Y-M, Owen D, Johnson CL (2007). Regulation of innate antiviral defenses through a shared repressor domain in RIG-I and LGP2.. Proceedings of the National Academy of Sciences.

[9] Iwasaki A, Medzhitov R (2004). Toll-like receptor control of the adaptive immune responses.. Nat Immunol.

[10] Alexopoulou L, Holt AC, Medzhitov R, Flavell RA (2001). Recognition of double-stranded RNA and activation of NF-kappaB by Toll-like receptor 3.. Nature.

[11] Diebold SS, Kaisho T, Hemmi H, Akira S, Reis e Sousa C (2004). Innate antiviral responses by means of TLR7-mediated recognition of single-stranded RNA.[see comment].. Science.

[12] Heil F, Hemmi H, Hochrein H, Ampenberger F, Kirschning C (2004). Species-specific recognition of single-stranded RNA via toll-like receptor 7 and 8.[see comment].. Science.

[13] Bauer S, Kirschning CJ, HÃ¤cker H, Redecke V, Hausmann S (2001). Human TLR9 confers responsiveness to bacterial DNA via species-specific CpG motif recognition.. Proceedings of the National Academy of Sciences of the United States of America.

[14] Andrejeva J, Childs KS, Young DF, Carlos TS, Stock N (2004). The V proteins of paramyxoviruses bind the IFN-inducible RNA helicase, mda-5, and inhibit its activation of the IFN-beta promoter.. Proceedings of the National Academy of Sciences of the United States of America.

[15] Rothenfusser S, Goutagny N, DiPerna G, Gong M, Monks BG (2005). The RNA helicase Lgp2 inhibits TLR-independent sensing of viral replication by retinoic acid-inducible gene-I.. Journal of Immunology.

[16] Sumpter R, Loo YM, Foy E, Li K, Yoneyama M (2005). Regulating intracellular antiviral defense and permissiveness to hepatitis C virus RNA replication through a cellular RNA helicase, RIG-I.. Journal of Virology.

[17] Yoneyama M, Kikuchi M, Matsumoto K, Imaizumi T, Miyagishi M (2005). Shared and unique functions of the DExD/H-box helicases RIG-I, MDA5, and LGP2 in antiviral innate immunity.. Journal of Immunology.

[18] Yoneyama M, Kikuchi M, Natsukawa T, Shinobu N, Imaizumi T (2004). The RNA helicase RIG-I has an essential function in double-stranded RNA-induced innate antiviral responses.[see comment].. Nature Immunology.

[19] Komuro A, Horvath CM (2006). RNA and Virus-Independent Inhibition of Antiviral Signaling by RNA Helicase LGP2.. Journal of Virology.

[20] Kang DC, Gopalkrishnan RV, Wu Q, Jankowsky E, Pyle AM (2002). mda-5: An interferon-inducible putative RNA helicase with double-stranded RNA-dependent ATPase activity and melanoma growth-suppressive properties.. Proceedings of the National Academy of Sciences of the United States of America.

[21] Kovacsovics M, Martinon F, Micheau O, Bodmer JL, Hofmann K (2002). Overexpression of Helicard, a CARD-containing helicase cleaved during apoptosis, accelerates DNA degradation.[erratum appears in Curr Biol. 2002 Sep 17;12(18):1633.].. Current Biology.

[22] Venkataraman T, Valdes M, Elsby R, Kakuta S, Caceres G (2007). Loss of DExD/H Box RNA Helicase LGP2 Manifests Disparate Antiviral Responses.. J Immunol.

[23] Kawai T, Takahashi K, Sato S, Coban C, Kumar H (2005). IPS-1, an adaptor triggering RIG-I- and Mda5-mediated type I interferon induction.[see comment].. Nature Immunology.

[24] Meylan E, Curran J, Hofmann K, Moradpour D, Binder M (2005). Cardif is an adaptor protein in the RIG-I antiviral pathway and is targeted by hepatitis C virus.. Nature.

[25] Seth RB, Sun L, Ea CK, Chen ZJ (2005). Identification and characterization of MAVS, a mitochondrial antiviral signaling protein that activates NF-kappaB and IRF 3.[see comment].. Cell.

[26] Xu LG, Wang YY, Han KJ, Li LY, Zhai Z (2005). VISA is an adapter protein required for virus-triggered IFN-beta signaling.. Molecular Cell.

[27] Hornung V, Ellegast J, Kim S, Brzozka K, Jung A (2006). 5′-Triphosphate RNA Is the Ligand for RIG-I.. Science.

[28] Pichlmair A, Schulz O, Tan CP, Naslund TI, Liljestrom P (2006). RIG-I-Mediated Antiviral Responses to Single-Stranded RNA Bearing 5′-Phosphates.. Science.

[29] Kato H, Takeuchi O, Mikamo-Satoh E, Hirai R, Kawai T (2008). Length-dependent recognition of double-stranded ribonucleic acids by retinoic acid-inducible gene-I and melanoma differentiation-associated gene 5.. J Exp Med.

[30] Saito T, Gale M (2008). Differential recognition of double-stranded RNA by RIG-I-like receptors in antiviral immunity.. J Exp Med.

[31] Saito T, Owen DM, Jiang F, Marcotrigiano J, Gale M (2008). Innate immunity induced by composition-dependent RIG-I recognition of hepatitis C virus RNA.. Nature.

[32] Gitlin L, Barchet W, Gilfillan S, Cella M, Beutler B (2006). Essential role of mda-5 in type I IFN responses to polyriboinosinic:polyribocytidylic acid and encephalomyocarditis picornavirus.. Proceedings of the National Academy of Sciences of the United States of America.

[33] Kato H, Takeuchi O, Sato S, Yoneyama M, Yamamoto M (2006). Differential roles of MDA5 and RIG-I helicases in the recognition of RNA viruses.. Nature.

[34] Loo Y-M, Fornek J, Crochet N, Bajwa G, Perwitasari O (2008). Distinct RIG-I and MDA5 Signaling by RNA Viruses in Innate Immunity.. J Virol.

[35] McCartney SA, Thackray LB, Gitlin L, Gilfillan S, Virgin Iv HW (2008). MDA-5 Recognition of a Murine Norovirus.. PLoS Pathog.

[36] Fredericksen BL, Keller BC, Fornek J, Katze MG, Gale M (2008). Establishment and Maintenance of the Innate Antiviral Response to West Nile Virus Involves both RIG-I and MDA5 Signaling through IPS-1.. J Virol.

[37] Collins PL, Chanock RM, McIntosh K, Fields (1996). Parainfluenza Viruses.. Fields Virology.

[38] Collins PL, Chanock RM, McIntosh K (1996). Respiratory Syncytial Virus. Fields Virology.

[39] Walter MJ, Kajiwara N, Karanja P, Castro M, Holtzman MJ (2001). Interleukin 12 p40 Production by Barrier Epithelial Cells during Airway Inflammation.. J Exp Med.

[40] Hausmann S, Marq J-B, Tapparel C, Kolakofsky D, Garcin D (2008). RIG-I and dsRNA-Induced IFNÎ^2^ Activation.. PLoS ONE.

[41] Strahle L, Marq J-B, Brini A, Hausmann S, Kolakofsky D (2007). Activation of the Beta Interferon Promoter by Unnatural Sendai Virus Infection Requires RIG-I and Is Inhibited by Viral C Proteins.. J Virol.

[42] Garcin D, Latorre P, Kolakofsky D (1999). Sendai Virus C Proteins Counteract the Interferon-Mediated Induction of an Antiviral State.. J Virol.

[43] Gotoh B, Takeuchi K, Komatsu T, Yokoo J, Kimura Y (1999). Knockout of the Sendai virus C gene eliminates the viral ability to prevent the interferon-[alpha]/[beta]-mediated responses.. FEBS Letters.

[44] Childs K, Stock N, Ross C, Andrejeva J, Hilton L (2007). mda-5, but not RIG-I, is a common target for paramyxovirus V proteins.. Virology.

[45] Yount JS, Gitlin L, Moran TM, Lopez CB (2008). MDA5 Participates in the Detection of Paramyxovirus Infection and Is Essential for the Early Activation of Dendritic Cells in Response to Sendai Virus Defective Interfering Particles.. J Immunol.

[46] Parker JC, Whiteman MD, Richter CB (1978). Susceptibility of inbred and outbred mouse strains to Sendai virus and prevalence of infection in laboratory rodents.. Infect Immun.

[47] Itoh T, Iwai H, Ueda K (1991). Comparative lung pathology of inbred strain of mice resistant and susceptible to Sendai virus infection.. Journal of Veterinary Medical Science.

[48] Mo XY, Sarawar SR, Doherty PC (1995). Induction of cytokines in mice with parainfluenza pneumonia.. J Virol.

[49] Blandford G, Heath RB (1972). Studies on the immune response and pathogenesis of Sendai virus infection of mice. I. The fate of viral antigens.. Immunology.

[50] Degré M, Midtvedt T (1971). Respiratory infection with parainfluenza 1, Sendai virus in gnotobiotic and conventional mice.. Acta pathologica et microbiologica Scandinavica Section B: Microbiology and immunology.

[51] Brownstein DGSA, Johnson EA (1981). Sendai virus infection in genetically resistant and susceptible mice.. The American journal of pathology.

[52] Takeuchi K, Komatsu T, Yokoo J, Kato A, Shioda T (2001). Sendai virus C protein physically associates with Stat1.. Genes to Cells.

[53] Shornick LP, Wells AG, Zhang Y, Patel AC, Huang G (2008). Airway Epithelial versus Immune Cell Stat1 Function for Innate Defense against Respiratory Viral Infection.. J Immunol.

[54] Lopez CB, Moltedo B, Alexopoulou L, Bonifaz L, Flavell RA (2004). TLR-Independent Induction of Dendritic Cell Maturation and Adaptive Immunity by Negative-Strand RNA Viruses.. J Immunol.

[55] Melchjorsen J, Jensen SB, Malmgaard L, Rasmussen SB, Weber F (2005). Activation of innate defense against a paramyxovirus is mediated by RIG-I and TLR7 and TLR8 in a cell-type-specific manner.. Journal of Virology.

[56] Kato A, Kiyotani K, Sakai Y, Yoshida T, Nagai Y (1997). The paramyxovirus, Sendai virus, V protein encodes a luxury function required for viral pathogenesis.. EMBO (European Molecular Biology Organization) Journal.

[57] Lei Y, Moore CB, Liesman RM, O'Connor BP, Bergstralh DT (2009). MAVS-Mediated Apoptosis and Its Inhibition by Viral Proteins.. PLoS ONE.

[58] Peters K, Chattopadhyay S, Sen GC (2008). IRF-3 Activation by Sendai Virus Infection Is Required for Cellular Apoptosis and Avoidance of Persistence.. J Virol.

[59] Kiyotani K, Sakaguchi T, Kato A, Nagai Y, Yoshida T (2007). Paramyxovirus Sendai virus V protein counteracts innate virus clearance through IRF-3 activation, but not via interferon, in mice.. Virology.

[60] Parisien J-P, Bamming D, Komuro A, Ramachandran A, Rodriguez JJ (2009). A Shared Interface Mediates Paramyxovirus Interference with Antiviral RNA Helicases MDA5 and LGP2.. J Virol.

